# The Role of Inflammatory Sarcopenia in Increasing Fall Risk in Older Adults: Exploring the Impact on Mobility-Impaired and Immunocompromised Patients

**DOI:** 10.3390/geriatrics10020052

**Published:** 2025-04-01

**Authors:** Marc-Dan Blajovan, Simona-Alina Abu-Awwad, Mirela-Cleopatra Tomescu, Cristina Tudoran, Daniela Gurgus, Anca Dinu, Ahmed Abu-Awwad

**Affiliations:** 1 Doctoral School, “Victor Babes” University of Medicine and Pharmacy, Eftimie Murgu Square, No. 2, 300041 Timisoara, Romania; marc.blajovan@umft.ro; 2 “Pius Brinzeu” Emergency Clinical County Hospital, Bld Liviu Rebreanu, No. 156, 300723 Timisoara, Romania; alina.abuawwad@umft.ro (S.-A.A.-A.); tudoran.cristina@umft.ro (C.T.); ahm.abuawwad@umft.ro (A.A.-A.); 3 Department XII, Discipline of Obstetrics and Gynecology, “Victor Babes” University of Medicine and Pharmacy, Eftimie Murgu Square, No. 2, 300041 Timisoara, Romania; 4 Multidisciplinary Heart Research Center, “Victor Babes” University of Medicine and Pharmacy, Eftimie Murgu Square, No. 2, 340001 Timisoara, Romania; tomescu.mirela@umft.ro; 5 Department of Internal Medicine I, Faculty of Medicine, “Victor Babes” University of Medicine and Pharmacy, Eftimie Murgu Square, No. 2, 340001 Timisoara, Romania; 6 Timisoara Municipal Clinical Emergency Hospital, Hector Str., Nr. 1, 300040 Timisoara, Romania; 7 Department VII, Internal Medicine II, Discipline of Cardiology, “Victor Babes” University of Medicine and Pharmacy, Eftimie Murgu Square, No. 2, 300041 Timisoara, Romania; 8 Center of Molecular Research in Nephrology and Vascular Disease, Faculty of the “Victor Babes” University of Medicine and Pharmacy, Eftimie Murgu Square, No. 2, 300041 Timisoara, Romania; 9 Department of Balneology, Medical Recovery and Rheumatology, Family Discipline, Center for Preventive Medicine, “Victor Babes” University of Medicine and Pharmacy, Eftimie Murgu Square, No. 2, 300041 Timisoara, Romania; 10 Department XVI—Medical Recovery, “Victor Babes” University of Medicine and Pharmacy, 300041 Timisoara, Romania; dinu.anca@umft.ro; 11 Research Center for Assessment of Human Motion and Functionality and Disability, “Victor Babes” University of Medicine and Pharmacy, Eftimie Murgu Square, No. 2, 300041 Timisoara, Romania; 12 Department XV—Discipline of Orthopedics—Traumatology, “Victor Babes” University of Medicine and Pharmacy, Eftimie Murgu Square, No. 2, 300041 Timisoara, Romania; 13 Research Center University Professor Doctor Teodor Șora, “Victor Babes” University of Medicine and Pharmacy, Eftimie Murgu Square, No. 2, 300041 Timisoara, Romania

**Keywords:** inflammatory sarcopenia, fall risk, systemic inflammation, CRP, IL-6, mobility impairment, immunocompromised adults

## Abstract

**Background/Objectives**: Inflammatory sarcopenia, characterized by muscle weakness exacerbated by chronic systemic inflammation, has emerged as a critical factor in fall risk among older adults. While previous studies have examined sarcopenia and inflammation independently, few have investigated their combined impact on mobility impairments and fall susceptibility, particularly in immunocompromised individuals. This study aimed to assess the role of inflammatory sarcopenia in increasing fall risk by comparing functional performance, muscle strength, and inflammatory biomarkers across three groups: healthy older adults, individuals with non-inflammatory sarcopenia, and those with inflammatory sarcopenia. A secondary objective was to evaluate fall incidence in immunocompromised versus non-immunocompromised individuals. **Methods**: A prospective observational study was conducted on 250 adults aged ≥65 years, categorized based on inflammatory status and muscle health. Functional assessments included handgrip strength, the Timed Up and Go (TUG) test, and fall frequency analysis. Inflammatory status was determined by measuring C-reactive protein (CRP) and interleukin-6 (IL-6) levels. Multivariate regression models were used to identify predictors of fall risk. **Results**: Participants with inflammatory sarcopenia exhibited significantly higher CRP and IL-6 levels, greater muscle weakness, poorer mobility performance, and a fourfold increase in fall incidence compared to controls (*p* < 0.001). Immunocompromised individuals had nearly double the fall risk of their non-immunocompromised counterparts (*p* < 0.001). TUG test performance was the strongest fall predictor. **Conclusions**: Our findings highlight the importance of integrating fall prevention strategies that not only focus on muscle-strengthening programs but also include regular screening for inflammatory markers. Given the strong association between systemic inflammation, muscle weakness, and fall risk, identifying and managing chronic inflammation may play a crucial role in reducing mobility impairments and improving outcomes in older adults.

## 1. Introduction

Aging is a natural process associated with various physiological changes, including the progressive loss of muscle mass and strength, a condition known as sarcopenia [1]. This phenomenon significantly contributes to frailty, decreased mobility, and an increased risk of falls among older adults [2]. Over the past decades, research has highlighted the role of chronic inflammation in the progression of sarcopenia, shaping the concept of inflammatory sarcopenia—a more severe form of the condition closely linked to immune dysfunction and persistent systemic inflammation [3].

Falls represent one of the leading causes of morbidity and mortality in older adults, with devastating consequences for their quality of life. Statistics indicate that approximately one-third of individuals over 65 experience at least one fall annually, with incidence rates increasing with age and comorbidities [4]. Major contributors to fall risk include muscle weakness, postural instability, and mobility impairments, all exacerbated by systemic inflammation [5].

Chronic inflammation plays a crucial role in accelerating muscle loss and neuromuscular dysfunction [6]. Inflammatory markers such as C-reactive protein (CRP) and interleukin-6 (IL-6) are frequently elevated in individuals with inflammatory sarcopenia and are strongly associated with progressive declines in muscle mass and function [7]. The mechanisms through which inflammation contributes to muscle deterioration include excessive activation of catabolic pathways, inhibition of muscle protein synthesis, and mitochondrial dysfunction. These processes lead to decreased muscle strength and reduced ability to perform daily activities, thereby increasing susceptibility to falls and injuries [8,9,10].

Although numerous studies have separately examined the impact of sarcopenia and inflammation on mobility and fall risk [11,12]; few have investigated their combined effects on frailty in older adults [13,14]. In this context, the present study aims to explore how inflammatory sarcopenia influences fall risk, focusing on immunocompromised individuals and those with mobility impairments.

This study provides a detailed perspective on how inflammation exacerbates muscle weakness and affects neuromotor function by categorizing participants into three distinct groups—healthy older adults, individuals with non-inflammatory sarcopenia, and those with inflammatory sarcopenia. Clinical assessments, including grip strength measurement, the Timed Up and Go (TUG) test, and fall frequency analysis, allow for the identification of specific risk factors associated with inflammatory sarcopenia. Furthermore, evaluating inflammatory biomarkers provides strong evidence of the link between systemic inflammation and physical performance decline.

Another key objective of this study is to investigate the impact of inflammatory sarcopenia on immunocompromised patients. Individuals with immune dysfunction exhibit elevated levels of systemic inflammation, which may accelerate muscle deterioration and increase vulnerability to falls. Comparing these individuals to non-immunocompromised counterparts may contribute to a deeper understanding of the interplay between immunity, inflammation, and mobility impairments.

Thus, this research seeks to answer a crucial question: To what extent does inflammation worsen the effects of sarcopenia on fall risk, and what strategies can be implemented to mitigate these negative effects? By addressing this question, our study contributes to a better understanding of the complexity of inflammatory sarcopenia and provides a foundation for developing effective prevention and treatment strategies.

## 2. Materials and Methods

### 2.1. Study Design and Population

This prospective study was conducted between 2022 and 2024. It aimed to investigate the role of inflammatory sarcopenia in increasing fall risk among older adults, with a particular focus on mobility-impaired and immunocompromised individuals.

The study population was older adults aged 65 and above, recruited from two healthcare facilities: the Outpatient Department of the Clinical Emergency County Hospital of Timișoara and the Orthopedics Medical Center. These institutions were selected to ensure a diverse patient cohort, including individuals with varying degrees of mobility impairment and inflammatory conditions.

Participants were categorized into three groups based on muscle health and inflammatory status: (1) healthy older adults (control group), (2) individuals with non-inflammatory sarcopenia, and (3) those with inflammatory sarcopenia, defined by the presence of both sarcopenia and elevated inflammatory markers (CRP > 6 mg/L and IL-6 > 5 pg/mL). Additionally, a secondary classification divided participants into immunocompromised and non-immunocompromised groups based on underlying medical conditions affecting immune function. These group classifications were independent of the general inclusion and exclusion criteria, which ensured a clinically relevant and homogeneous population for analysis.

Participants were enrolled based on specific inclusion and exclusion criteria. The inclusion criteria were:

- Individuals aged 65 years and older, ensuring a population at higher risk for sarcopenia and falls.
- Participants who can walk at least 10 m independently, with or without assistive devices, to allow for functional mobility assessments.
- Individuals without acute or uncontrolled medical conditions that could interfere with muscle function or inflammatory status (e.g., no hospitalization for acute illness in the past three months).
- Sarcopenia Classification—Sarcopenia was diagnosed based on the criteria established by EWGSOP2 (European Working Group on Sarcopenia in Older People); which provides standardized definitions for sarcopenia assessment; including muscle strength; mass, and physical performance [11].
- Inflammatory Marker Availability—Blood tests for C-reactive protein (CRP) and interleukin-6 (IL-6) must be available for classification into inflammatory and non-inflammatory sarcopenia subgroups.
- Cognitive Competence—Participants must have preserved cognitive function (Mini-Mental State Examination [MMSE] ≥ 18); ensuring their ability to understand and complete the study procedures [15].
- Informed Consent—Must provide signed informed consent; demonstrating willingness to participate and comply with study protocols.

Exclusion criteria included:

- Neurological Disorders Affecting Mobility—Diagnosis of stroke; Parkinson’s disease; multiple sclerosis; amyotrophic lateral sclerosis (ALS); or other progressive neurological conditions that independently impair muscle function and mobility.
- Musculoskeletal Disorders—Presence of severe osteoarthritis; recent fractures; limb amputations; or advanced spinal conditions that significantly limit movement and physical testing.
- Uncontrolled Chronic Diseases—Unstable heart failure, severe chronic obstructive pulmonary disease (COPD); end-stage renal disease; or other conditions likely to cause functional decline independent of sarcopenia.
- Active Malignancy—Current cancer diagnosis or ongoing chemotherapy/radiotherapy within the past six months; as these conditions may significantly alter inflammatory status.
- Use of High-Dose Immunosuppressants—Chronic use of high-dose corticosteroids; biologic agents; or cytotoxic immunosuppressive drugs that could confound inflammation-related outcomes.
- History of Recent Falls Due to External Factors—Individuals whose falls were primarily caused by environmental hazards or external trauma (e.g., road accidents, falls from height, intoxication-related falls) rather than intrinsic physical decline.
- Severe Sensory Impairment—Profound visual or vestibular dysfunction that independently affects balance and increases fall risk beyond musculoskeletal factors.

The immunocompromised cohort included participants with mild to moderate immune dysfunction, excluding those on high-dose immunosuppressants or with active malignancies. An immune deficiency was attributed to age-related immunosenescence, low-dose corticosteroid use (≤5 mg/day), type 2 diabetes with HbA1c ≥ 7.5%, chronic kidney disease (stages 3–4), and a history of cancer in remission (>6 months). These conditions are known to impair immune function and contribute to chronic inflammation, aligning with the study’s focus on inflammatory sarcopenia.

By adhering to strict exclusion criteria, we ensured that immunocompromised participants had immune alterations without confounding effects from severe immunosuppression or unstable disease, allowing for a precise assessment of their muscle function and fall risk.

By applying these well-defined criteria, the study ensured the inclusion of a clinically relevant and homogenous population while minimizing confounding variables that could interfere with evaluating inflammatory sarcopenia and fall risk.

### 2.2. Data Collection

Data collection for this study was conducted prospectively between 2022 and 2024 at the Outpatient Department of the Clinical Emergency County Hospital of Timișoara and the Orthopedics Medical Center. Upon enrollment, all participants underwent a comprehensive assessment, including demographic, clinical, biochemical, and functional evaluations. Data were recorded at baseline and during follow-up visits to track changes in muscle function, inflammatory status, and fall incidence.

Demographic data, including age, sex, body mass index (BMI), medical history, medication use, and comorbidities, were obtained through structured interviews and medical records review. Special attention was given to conditions known to influence muscle function and inflammation, such as diabetes, cardiovascular disease, rheumatoid arthritis, and chronic kidney disease.

Inflammatory status was assessed through blood sample analysis, measuring key inflammatory biomarkers, including CRP, IL-6, TNF-α, and fibrinogen. Among these, CRP and IL-6 showed the strongest associations with muscle function and fall risk, making them the primary focus of the analysis. Blood samples were collected after an overnight fast and processed at the hospital’s clinical laboratory using standardized protocols. Patients with elevated inflammatory markers were classified as having inflammatory sarcopenia, while those with normal levels were assigned to the non-inflammatory sarcopenia or control groups, depending on their muscle health.

Muscle strength and functional performance were evaluated using objective physical assessments. Handgrip strength was measured using a dynamometer, with three trials conducted on the dominant hand, and the highest value was recorded. Mobility was assessed through the Timed Up and Go (TUG) test, where participants were instructed to rise from a chair, walk three meters, turn around, and return to the seated position. Gait speed was measured using the 4-m walking test, and balance was evaluated through standardized postural stability tests. Trained physiotherapists performed these assessments to ensure consistency and reliability.

Fall incidence data were collected through patient interviews and medical records review. Participants were asked to report any falls experienced in the past 12 months, including details on frequency, circumstances, and any resulting injuries. Follow-up assessments were conducted every six months to monitor new fall events and functional changes. Any reported falls were verified through caregiver interviews and hospital records when available.

To ensure data quality, all assessments were conducted using standardized protocols, and interobserver reliability was maintained through periodic training sessions for clinical staff. Data were securely stored in an encrypted database, accessible only to authorized researchers, in compliance with ethical and confidentiality standards. This structured data collection approach allowed for a comprehensive analysis of the relationship between inflammatory sarcopenia, functional decline, and fall risk in older adults.

To ensure consistency in data collection, all participants followed an identical follow-up protocol, with scheduled assessments every six months. During each visit, muscle function, mobility, inflammatory markers (CRP, IL-6), and fall incidence were reassessed using the same standardized methods as at baseline. Evaluations were conducted by trained clinicians following a predefined protocol, ensuring uniformity across all participants. Additionally, fall events were verified through patient interviews, caregiver reports, and medical records, where available. This standardized approach minimized variability and enhanced the reliability of our findings.

### 2.3. Statistical Analysis

Statistical analyses were performed using GraphPad Prism 6, ensuring rigorous evaluation of the collected data. A significance level of *p* < 0.05 was considered statistically significant for all tests. Descriptive statistics were first used to summarize participant characteristics, including means and standard deviations (SD) for continuous variables (e.g., age, BMI, handgrip strength) and frequencies with percentages for categorical variables (e.g., sex, fall incidence).

Between-group comparisons were conducted using one-way analysis of variance (ANOVA) for normally distributed continuous variables, followed by post-hoc Bonferroni or Tukey tests when significant differences were identified. For non-normally distributed data, the Kruskal–Wallis test was applied. Categorical variables, such as fall incidence across groups, were analyzed using the chi-square (χ^2^) test or Fisher’s exact test, depending on sample size and distribution.

To assess the relationship between inflammatory markers (CRP, IL-6), muscle strength (handgrip test), and mobility performance (Timed Up and Go [TUG] test, walking speed), Pearson’s correlation coefficient (r) was used for normally distributed data, while Spearman’s rank correlation was employed for non-parametric data.

Multivariate analysis was performed using logistic regression models to identify independent predictors of fall risk. The dependent variable was fall occurrence (yes/no), while independent variables included inflammatory markers, muscle strength, mobility performance, and demographic factors. Odds ratios (ORs) with 95% confidence intervals (CI) were reported to quantify the strength of association. Additionally, multiple linear regression was conducted to evaluate the impact of inflammation on muscle function and mobility scores while adjusting for potential confounders such as age, sex, and BMI.

To further explore fall risk prediction, receiver operating characteristic (ROC) curve analysis was performed to assess the discriminatory power of inflammatory markers and functional tests in predicting falls. The area under the curve (AUC) was calculated, with higher AUC values indicating better predictive ability.

Finally, subgroup analyses were conducted to compare immunocompromised vs. non-immunocompromised participants, using independent *t*-tests (or Mann–Whitney U tests for non-parametric data) to determine differences in inflammatory status, muscle strength, and fall rates.

All statistical tests were two-tailed, and results were interpreted considering both statistical significance and clinical relevance. The comprehensive use of parametric and non-parametric methods ensured a robust evaluation of the interplay between inflammation, sarcopenia, mobility impairment, and fall risk in older adults.

### 2.4. Ethical Considerations

This study was conducted in full compliance with the ethical principles outlined in the Declaration of Helsinki (2013 revision). It was approved by the Ethics Committee of the Clinical Emergency County Hospital of Timișoara (Approval No. 106/12.12.2021). and the Orthopedics Medical Center Ethics Board (Approval No. 5/23.11.2021). All participants provided written informed consent before enrollment, ensuring their voluntary participation and understanding of the study objectives, procedures, potential risks, and benefits.

Confidentiality and data protection were strictly maintained throughout the study. Personal information and medical data were anonymized and stored in a secure, encrypted database accessible only to authorized research personnel. All collected data were used solely for research purposes and were not disclosed to third parties.

Participants were informed of their right to withdraw from the study at any time without any consequences regarding their medical care. Additionally, any participants identified as having a significantly increased fall risk or severe sarcopenia were referred to appropriate medical services for further assessment and intervention.

Given the study’s focus on older and potentially vulnerable individuals, special attention was given to ensuring that participants fully understood the study’s scope. For individuals with mild cognitive impairment, a legally authorized representative was consulted before obtaining consent.

No invasive procedures were performed beyond routine blood sampling, and all physical assessments (e.g., handgrip strength, TUG test) were non-invasive and conducted under supervised clinical conditions to minimize the risk of injury. The study protocol was periodically reviewed to ensure adherence to ethical guidelines and patient safety.

Any modifications to the study design or procedures were submitted for reevaluation by the Ethics Committee, ensuring ongoing compliance with ethical and legal standards.

## 3. Results

### 3.1. Study Population and Group Classification

The study included a total of 250 older adults aged 65 and above, categorized into three distinct groups based on their muscle health and inflammatory status. The first group (CG) (*n* = 80) consisted of healthy controls with no signs of sarcopenia, serving as a reference for comparison. The second group (*n* = 85) included individuals with non-inflammatory sarcopenia (SC), characterized by muscle weakness and reduced mass but without elevated inflammatory markers. Finally, the third group (*n* = 85) comprised participants with inflammatory sarcopenia (ISG), who exhibited both muscle deterioration and heightened systemic inflammation. This classification allowed for a detailed analysis of how inflammation contributes to sarcopenia progression and, more importantly, how it influences fall risk and mobility impairment in aging individuals.

The study groups showed notable differences in body composition, inflammatory markers, muscle strength, mobility, and fall incidence, despite similar age and sex distribution (Table 1). Participants with sarcopenia, especially those with inflammation, had lower BMI and significantly higher CRP and IL-6 levels, highlighting the role of chronic inflammation in muscle deterioration. Muscle strength declined progressively across groups, with the inflammatory sarcopenia group showing the most significant weakness and mobility impairment, as evidenced by poorer handgrip strength and slower Timed Up-and-Go (TUG) test performance. Most notably, fall incidence was over four times higher in the inflammatory sarcopenia group compared to controls, reinforcing the link between inflammation, sarcopenia, and fall risk.

The inflammatory sarcopenia group had a significantly higher prevalence of hypertension, diabetes, cardiovascular disease, and chronic kidney disease compared to the other groups. Additionally, osteoporosis and osteoarthritis were more frequent among sarcopenic individuals, particularly those with inflammation. Depression was also notably more common in participants with inflammatory sarcopenia, suggesting a potential link between systemic inflammation and mental health decline. Furthermore, a history of falls was markedly higher in sarcopenic individuals, especially in the inflammatory subgroup, supporting the study hypothesis that inflammation exacerbates fall risk.

### 3.2. Functional Performance and Fall Risk

Functional performance and fall risk vary significantly across the study groups, with an apparent decline observed in individuals with sarcopenia, particularly those with an inflammatory profile. Mobility impairments become progressively more pronounced, as evidenced by slower performance on the Timed Up and Go (TUG) test and reduced walking speed. Postural stability is also significantly compromised, with a notable decrease in balance test success rates among participants with inflammatory sarcopenia. The trends presented in Table 2 indicate that inflammation exacerbates muscle weakness and negatively impacts coordination and mobility, increasing overall fall risk. The statistically significant differences across groups confirm the strong association between inflammatory status, functional decline, and fall susceptibility in older adults.

The regression analysis identifies key independent predictors of fall risk in older adults, demonstrating a strong association between systemic inflammation, muscle weakness, and impaired mobility. Elevated inflammatory markers significantly increase the likelihood of falls, reinforcing the role of chronic inflammation in exacerbating frailty. Reduced muscle strength is also critical, suggesting that diminished handgrip power reflects overall musculoskeletal decline. Additionally, poor mobility, as indicated by prolonged TUG test performance, is the strongest predictor, highlighting the importance of gait and functional assessments in fall risk evaluation. Table 3 confirms that multiple physiological impairments contribute to fall susceptibility, emphasizing the need for targeted interventions addressing inflammation and functional deficits.

### 3.3. Immunocompromised Status, Inflammation, and Fall Susceptibility

The data from Table 4 illustrate a strong relationship between systemic inflammation, muscle function, mobility, and fall risk, revealing both direct and inverse correlations. Higher inflammatory marker levels are consistently associated with poorer physical performance, indicating that increased systemic inflammation contributes to muscle weakness, reduced walking speed, and impaired balance. The negative correlations with muscle strength and balance suggest that as inflammation rises, functional capacity declines, while the positive correlation with mobility impairment confirms that inflammation exacerbates movement difficulties. The statistical significance of all associations reinforces the idea that inflammatory processes play a central role in functional decline, further supporting their inclusion as key indicators in fall risk assessments.

Table 5 illustrates significant differences between individuals who experienced falls and those who did not, highlighting key physiological and inflammatory factors associated with fall risk. Older adults with a history of falls exhibited higher levels of systemic inflammation, weaker muscle strength, and impaired mobility, suggesting a direct link between chronic inflammation, functional decline, and fall susceptibility. The observed disparities in physical performance indicate that reduced muscular capacity and slower movement speed contribute to postural instability and an increased likelihood of falling. The statistical significance of these differences further supports the role of inflammation and functional impairments as critical determinants of fall risk in aging populations.

A progressive increase in fall frequency is evident as sarcopenia worsens and systemic inflammation becomes more pronounced (Table 6). Individuals with muscle loss but without significant inflammation show a moderate rise in fall incidence, whereas those with inflammatory sarcopenia experience a substantially higher rate of recurrent falls. This pattern underscores the amplifying effect of inflammation on fall risk, suggesting that chronic inflammatory processes further weaken muscle function, impair balance, and reduce overall stability. The statistically significant differences between groups indicate that inflammatory sarcopenia constitutes a distinct high-risk condition, with a markedly greater likelihood of multiple falls compared to both healthy individuals and those with non-inflammatory sarcopenia.

Multivariate regression analysis identifies several independent predictors of fall risk, with systemic inflammation, muscle weakness, and impaired mobility emerging as significant contributing factors. Higher levels of inflammatory markers are strongly associated with an increased likelihood of falling, reinforcing the role of chronic inflammation in accelerating functional decline. Reduced muscle strength is also a key determinant, highlighting the impact of sarcopenia on stability and fall susceptibility. Poor performance on mobility assessments further amplifies risk, with prolonged TUG test times showing the strongest predictive value. A history of previous falls remains a critical factor, suggesting that fall recurrence is influenced by both past incidents and underlying physiological deficits. Table 7 confirms that fall risk is multifactorial, emphasizing the need for integrated prevention strategies targeting inflammation, muscle preservation, and mobility enhancement in older adults.

A comparison between immunocompromised and non-immunocompromised older adults reveals notable differences in inflammation levels, muscle strength, mobility, and fall incidence. Individuals with immune dysfunction exhibit significantly higher inflammatory marker levels, suggesting a stronger systemic inflammatory response that may contribute to accelerated muscle deterioration. Reduced handgrip strength in this subgroup indicates greater muscular impairment, which, combined with poorer TUG test performance, points to more pronounced mobility limitations. The substantially higher fall incidence further underscores the increased vulnerability of immunocompromised individuals, highlighting the compounded effect of inflammation and muscle weakness on fall risk. Table 8 supports the hypothesis that immune status plays an important role in determining functional decline and fall susceptibility in aging populations.

The increased fall incidence observed in immunocompromised individuals (Table 8) aligns with the key fall risk predictors identified in Table 7, particularly elevated inflammatory markers (CRP > 6 mg/L, IL-6 > 5 pg/mL), reduced handgrip strength (<25 kg), and prolonged TUG test time (>12 s). These findings support the role of systemic inflammation and muscle weakness as major contributors to fall risk in this population.

## 4. Discussion

This study provides new insights into the complex interplay between inflammation, sarcopenia, and fall risk, reinforcing previous findings while refining the classification of sarcopenia based on inflammatory status. Unlike prior research that examined sarcopenia and inflammation as separate entities [16], our results emphasize their synergistic effect on muscle function and mobility decline. The progressive deterioration observed in BMI, handgrip strength, and TUG test performance across study groups suggests that inflammation accelerates muscle loss and functional impairment, making individuals with inflammatory sarcopenia particularly susceptible to falls.

The pathophysiological mechanisms underlying this relationship involve a combination of chronic systemic inflammation, muscle degradation, and neuromuscular dysfunction [6]. Elevated levels of proinflammatory cytokines such as IL-6 and CRP activate catabolic pathways, promoting muscle protein breakdown while inhibiting muscle synthesis. This contributes to a gradual decline in muscle mass and strength, further compounded by mitochondrial dysfunction and oxidative stress, which impair energy production and muscle endurance [17]. Additionally, inflammation interferes with neuromuscular function, leading to slower motor unit firing rates and reduced coordination, negatively impacting balance and gait stability [18]. These physiological changes collectively contribute to fall susceptibility, reinforcing the need for interventions addressing muscle preservation and reducing inflammation in older adults.

One of the most notable findings in this study is the strong association between inflammatory markers (CRP and IL-6) and fall risk, which remains statistically significant even after adjusting for muscle strength and mobility. While previous research has suggested a link between chronic inflammation and muscle degradation [19], our findings demonstrate its direct clinical impact, with fall incidence being over four times higher in the inflammatory sarcopenia group compared to controls. This underscores the limitations of traditional fall risk assessment models, which often rely solely on muscle mass or strength measurements without considering inflammatory status. Incorporating inflammatory markers as predictive variables could enhance the early identification of high-risk individuals and facilitate more effective prevention strategies.

A key contribution of this study is the direct comparison between inflammatory and non-inflammatory sarcopenia, offering a more detailed perspective on how inflammation modulates sarcopenia progression. Our findings suggest that interventions combining anti-inflammatory strategies with muscle-strengthening programs may be more effective in reducing fall risk than conventional sarcopenia treatments focused solely on muscle mass preservation. Distinguishing inflammatory sarcopenia as a high-risk condition allows for a more precise understanding of frailty mechanisms, paving the way for personalized fall prevention strategies tailored to inflammatory status [20,21,22].

The presence of comorbidities such as hypertension, diabetes, cardiovascular disease, chronic kidney disease, and osteoporosis was significantly higher in individuals with sarcopenia, particularly in those with systemic inflammation. These conditions contribute to persistent inflammation, oxidative stress, and metabolic dysregulation, all of which accelerate muscle degradation and functional decline [23,24,25,26,27,28]. Additionally, osteoporosis and osteoarthritis, which were more frequent among sarcopenic individuals, further impaired mobility and postural stability, increasing the risk of falls and fractures [29].

The higher prevalence of depression in the inflammatory sarcopenia group suggests a bidirectional link between chronic inflammation, neuromuscular decline, and mental health deterioration. Psychological distress may lead to reduced physical activity and increased frailty, exacerbating muscle weakness and balance deficits [30]. These findings emphasize the need for a multidimensional approach in sarcopenia management, addressing both inflammation control and comorbidity management to reduce fall risk in older adults.

Our results demonstrate an evident decline in functional performance as sarcopenia progresses, with the most severe impairments observed in individuals with systemic inflammation. These limitations are apparent in significantly slower TUG test performance and reduced walking speed, which are strong indicators of fall risk in aging populations. Postural stability is also compromised, with a marked decrease in balance test success rates, further increasing fall susceptibility.

While previous studies have explored the relationship between muscle loss and mobility deficits, fewer have quantified the additive impact of systemic inflammation [3,31]. Our findings provide stronger evidence that inflammation exacerbates neuromuscular impairments, making it a distinct contributor to functional decline. The cumulative effect of muscle weakness, impaired coordination, and increased inflammatory burden creates a high-risk environment for falls, reinforcing the need for targeted interventions.

Multivariate analysis identified systemic inflammation, muscle weakness, and impaired mobility as strong, independent predictors of fall risk. Elevated CRP and IL-6 levels significantly increased fall likelihood, reinforcing the idea that inflammation accelerates muscle degradation and neuromuscular dysfunction, leading to postural instability.

Additionally, handgrip strength, a well-established marker of musculoskeletal decline [32], emerged as a reliable predictor of fall risk, supporting previous findings. Among all analyzed variables, prolonged TUG test performance was the strongest predictor, emphasizing its clinical relevance in fall risk assessments. While prior studies have examined these factors separately, our study integrates them into a multivariate model, demonstrating their combined effects on fall susceptibility.

These results highlight the need for multidimensional fall prevention strategies that incorporate inflammation control, muscle preservation, and mobility enhancement—an approach that remains underrepresented in current clinical guidelines.

A particularly concerning finding is the significantly higher fall incidence among immunocompromised older adults [33], who exhibited elevated systemic inflammation, greater muscle weakness, and worse mobility performance compared to their non-immunocompromised counterparts [34]. Higher CRP and IL-6 levels in this group align with prior research linking immune dysfunction to muscle wasting and reduced physical resilience [35].

The nearly twofold increase in fall incidence among immunocompromised individuals [33] suggests that immunosenescence and persistent inflammation create a high-risk environment for functional decline and instability [36]. These findings reinforce the need for tailored interventions that address both inflammatory control and muscle preservation in this vulnerable population [37]. Incorporating immune status assessment into fall risk screening protocols may help identify high-risk individuals earlier and improve preventive strategies [38].

The findings of this study suggest that fall prevention strategies should not be limited to traditional muscle-strengthening programs but should also incorporate anti-inflammatory approaches [16]. Given the strong predictive value of inflammatory markers, future research should explore targeted anti-inflammatory therapies, such as pharmacological interventions, dietary modifications, and structured exercise programs aimed at reducing systemic inflammation [39].

Additionally, the integration of biomarkers like CRP and IL-6 into clinical practice could enhance early detection of high-risk individuals, allowing for more personalized interventions [40]. Future studies should investigate the long-term impact of inflammation-reducing therapies on sarcopenia progression and fall risk, ideally through randomized controlled trials [41]. Understanding whether lowering systemic inflammation can improve muscle function and mobility will be critical in developing comprehensive frailty management programs for older adults [42].

### Strengths, Limitations, and Novel Contributions of This Study

This study presents several notable strengths that enhance its scientific and clinical relevance. First, the inclusion of a well-defined cohort of 250 older adults, categorized based on both sarcopenia status and inflammatory markers, allows for a detailed examination of the interplay between muscle deterioration, systemic inflammation, and fall risk. Unlike prior research that has largely investigated sarcopenia and inflammation as independent factors, this study provides a direct comparison between inflammatory and non-inflammatory sarcopenia, demonstrating that inflammation exacerbates mobility impairments and fall susceptibility beyond the effects of muscle loss alone. Additionally, the study employs a comprehensive set of functional assessments (e.g., TUG test, grip strength, balance testing), providing a robust evaluation of physical performance in relation to inflammatory status. The use of multivariate regression models further strengthens the findings by controlling for potential confounders, allowing for a more precise identification of independent fall risk factors.

The findings of this study could have significant clinical implications. They emphasize the need for personalized fall prevention strategies that combine muscle-strengthening exercises with anti-inflammatory interventions. Identifying inflammatory markers as predictors of falls could facilitate the early detection of high-risk individuals and guide the development of more effective therapies to preserve mobility and independence in older adults.

Despite these strengths, several limitations should be acknowledged. The cross-sectional design prevents the establishment of causal relationships between inflammation, sarcopenia progression, and fall risk, necessitating longitudinal studies to confirm whether reducing inflammation can effectively mitigate falls. Additionally, the study does not account for potential confounders such as medication use, nutritional status, or pre-existing comorbidities, which could influence both inflammation levels and physical performance. Another limitation is the lack of direct mechanistic investigation, such as muscle biopsy or inflammatory pathway analysis, which could have provided deeper insights into the biological mechanisms linking inflammation and functional decline. Moreover, while the study highlights the increased fall risk in immunocompromised individuals, it does not differentiate between specific immunosuppressive conditions, limiting its generalizability to diverse clinical populations.

This study contributes several novel insights to the existing literature. It is among the first to define inflammatory sarcopenia as a distinct high-risk phenotype for falls, demonstrating that elevated CRP and IL-6 levels significantly amplify functional impairments. The results suggest that fall risk prediction models should incorporate inflammatory markers, rather than relying solely on traditional sarcopenia assessments. Additionally, the study underscores the particular vulnerability of immunocompromised older adults, an aspect often overlooked in fall prevention research. By highlighting the synergistic effects of inflammation and muscle weakness, these findings pave the way for integrated interventions combining anti-inflammatory strategies with resistance training, which could be more effective in reducing fall incidence than conventional sarcopenia management alone.

## 5. Conclusions

This study demonstrates that inflammatory sarcopenia is a significant and independent predictor of fall risk in older adults, particularly among those with mobility impairments and immunocompromised conditions. Elevated C-reactive protein and interleukin-6 were strongly correlated with reduced muscle strength, slower mobility, and increased fall incidence. Patients with inflammatory sarcopenia had nearly three times higher odds of falling compared to those without systemic inflammation. These findings highlight a potential inflammatory pathway contributing to sarcopenia-related falls.

Older adults with inflammatory sarcopenia exhibited significant functional impairments, including weaker grip strength and slower walking speeds. They prolonged Timed Up and Go test performance—all well-established markers of fall risk. Balance impairment was most severe in this group, with only 52% completing a standardized balance test, compared to 96% in the control group.

Furthermore, our findings suggest that immunocompromised individuals face an even greater risk, exhibiting higher inflammatory marker levels, weaker muscle function, and nearly double the fall incidence compared to non-immunocompromised participants. These results underscore the importance of targeted fall prevention strategies in high-risk populations.

Given the strong association between inflammation, sarcopenia, and fall risk, future interventions should consider anti-inflammatory treatments, resistance training, and early screening strategies to mitigate fall-related injuries. Monitoring inflammatory markers could provide a novel approach to identifying individuals at the highest risk. Addressing inflammation may be crucial in preventing falls and improving mobility in older adults.

## Figures and Tables

**Table 1:** Demographic and clinical characteristics of the study groups.

Characteristic		CG	SG	ISG	*p*-Value
Age (years)		72.4 ± 4.5	73.1 ± 5.1	74.2 ± 4.8	0.055
Sex	M	38 (47.5%)	41 (48.23%)	39 (45.88%)	0.721
	F	42 (52.5%)	44 (51.76%)	46 (54.11%)	0.651
BMI (kg/m^2^)		24.8 ± 3.1	23.5 ± 2.8	22.3 ± 2.9	<0.001
C-reactive protein (mg/L)		2.1 ± 1.3	4.8 ± 2.5	8.6 ± 3.1	<0.001
IL-6 (pg/mL)		1.9 ± 0.9	3.7 ± 1.5	6.5 ± 2.3	<0.001
Handgrip strength (kg)		32.4 ± 5.2	26.3 ± 4.9	21.1 ± 5.4	<0.001
TUG test (s)		8.1 ± 1.2	11.5 ± 2.4	14.2 ± 3.1	<0.001
Falls in the last 12 months		8 (10%)	21 (24.70%)	38 (44.7%)	<0.001
Comorbidities					
Hypertension		45 (56.25%)	58 (68.23%)	64 (75.29%)	0.02
Type 2 Diabetes Mellitus		12 (15%)	27 (31.76%)	39 (45.88%)	<0.001
Chronic Kidney Disease		7 (8.75%)	16 (18.82%)	29 (34.11%)	<0.001
CVD		19 (23.75%)	31 (36.47%)	46 (54.11%)	<0.001
COPD		6 (7.5%)	14 (16.47%)	28 (32.94%)	<0.001
Osteoarthritis		22 (27.5%)	37 (43.52%)	51 (60%)	<0.001
RA		3 (3.75%)	9 (10.58%)	18 (21.17%)	0.004
Osteoporosis		11 (13.75%)	26 (30.58%)	43 (50.58%)	<0.001
Depression		3 (3.75%)	14 (16.47%)	31 (36.47%)	<0.001

(M = Male, F = Female, BMI = Body Mass Index; IL-6 = Interleukin-6; TUG = Timed Up and Go Test, CVD = Cardiovascular Disease, COPD = Chronic Obstructive Pulmonary Disease, RA = Rheumatoid Arthritis).

**Table 2:** Functional performance and fall risk assessment.

Functional Test	CG	SG	ISG	*p*-Value
TUG (s)	8.1 ± 1.2	11.5 ± 2.4	14.2 ± 3.1	<0.001
4-m walking test	1.2 ± 0.2 m/s	0.9 ± 0.1 m/s	0.7 ± 0.2 m/s	<0.001
Balance test success	96%	78%	52%	<0.001

**Table 3:** Multivariate regression analysis for fall risk.

Predictor Variable	OR (95% CI)	*p*-Value
CRP > 6 mg/L	2.12 (1.54–3.01)	<0.001
IL-6 > 5 pg/mL	2.85 (1.91–4.23)	<0.001
Reduced handgrip strength	1.76 (1.32–2.44)	0.002
TUG > 12 s	3.14 (2.07–4.62)	<0.001

**Table 4:** Inflammatory markers and their correlation with functional performance and fall risk.

Variable	CRP (mg/L) (r)	IL-6 (pg/mL) (r)	*p*-Value
Handgrip Strength (kg)	−0.58	−0.62	<0.001
TUG Test (s)	0.61	0.67	<0.001
4-Meter Walk Speed (m/s)	−0.54	−0.59	<0.001
Balance Test Score	−0.45	−0.48	<0.001

**Table 5:** Fall incidence based on inflammatory and functional parameters.

Parameter	Falls (*n* = 67)	No Falls (*n* = 183)	*p*-Value
Age (years)	74.5 ± 4.2	72.8 ± 4.7	0.02
CRP (mg/L)	7.4 ± 2.9	3.9 ± 2.1	<0.001
IL-6 (pg/mL)	6.2 ± 2.5	3.1 ± 1.8	<0.001
Handgrip Strength (kg)	21.5 ± 5.3	29.1 ± 4.7	<0.001
TUG (s)	13.8 ± 2.9	9.7 ± 2.1	<0.001

**Table 6:** Fall frequency by sarcopenia and inflammatory status.

Group	No Falls (%)	1 Fall (%)	2 Falls (%)	*p*-Value
CG (*n* = 80)	72 (90%)	6 (7.5%)	2 (2.5%)	<0.001
SG (*n* = 85)	64 (75.3%)	12 (14.1%)	9 (10.6%)	<0.001
ISG (*n* = 85)	47 (55.3%)	15 (17.6%)	23 (27.1%)	<0.001
*p*-Value	<0.001	<0.001	<0.001	

**Table 7:** Regression model for fall risk prediction (Adjusted for covariates).

Variable	OR (95% CI)	*p*-Value
Age (per year)	1.09 (1.02–1.17)	0.01
CRP > 6 mg/L	2.45 (1.58–3.79)	<0.001
IL-6 > 5 pg/mL	3.02 (2.01–4.53)	<0.001
Handgrip Strength < 25 kg	2.10 (1.46–3.12)	<0.001
TUG > 12 s	3.32 (2.14–5.14)	<0.001
History of previous fall	2.74 (1.89–3.99)	<0.001

**Table 8:** Subgroup Analysis: Fall risk in immunocompromised vs. non-immunocompromised older adults.

Variable	Immunocompromised (*n* = 60)	Non-Immunocompromised (*n* = 190)	*p*-Value
CRP (mg/L)	7.9 ± 3.2	4.2 ± 2.5	<0.001
IL-6 (pg/mL)	6.7 ± 2.4	3.9 ± 1.8	<0.001
Handgrip Strength (kg)	21.3 ± 5.1	27.5 ± 4.9	<0.001
TUG (s)	14.5 ± 2.7	10.8 ± 2.3	<0.001
Fall Incidence (%)	48%	26%	<0.001

## Data Availability

The datasets used and analyzed during the current study are available from the first author.
